# Post-traumatic hydrocephalus in adults: the mechanisms of development, predictors of progression and management strategies. A narrative review and case series analysis

**DOI:** 10.3389/fneur.2026.1823124

**Published:** 2026-06-24

**Authors:** Bruno Splavski, Dario Muzevic

**Affiliations:** 1 Department of Neurosurgery, Dubrovnik General Hospital, Dubrovnik, Croatia; 2 University of Applied Health Sciences, Zagreb, Croatia; 3 Department of Neurosurgery, University Hospital Center Sestre Milosrdnice, Zagreb, Croatia; 4 European Academy of Sciences and Arts, Salzburg, Austria; 5 Department of Neurosurgery, Osijek University Hospital Center, Osijek, Croatia; 6 Faculty of Medicine, University of Osijek, Osijek, Croatia

**Keywords:** management strategies, mechanisms, post-traumatic hydrocephalus, progression predictors, traumatic brain injury

## Abstract

**Background:**

Post-traumatic hydrocephalus (PTH) is a common but dangerous complication of traumatic brain injury (TBI), increasing its morbidity/mortality and complicating the outcomes. It may arise abruptly or appear later following injury, and it is sometimes linked to substantial ventriculomegaly. Its etiology is complex and multifactorial, including severe TBI, subarachnoid/intracranial/intraventricular hemorrhage, and decompressive craniectomy. However, the exact mechanisms of PTH development, including cerebrospinal fluid (CSF) disturbances, supported by neuroinflammation and subarachnoid obstruction, remain inadequately determined. Consequently, managing PTH patients is demanding and logistically challenging, involving detailed diagnostics, external or V-P shunting, regular follow-ups, and treatment of complications such as CSF leaks, shunt malfunctions, obstructions, over-drainage, and infections.

**Methods:**

This narrative review aims to discuss and examine the mechanisms of PTH development in adults, predictors of its outcome/progression, and the modalities and effectiveness of its surgical management. It is based on a comprehensive review of the relevant literature and a single-center retrospective analysis of a cohort of adult PTH patients over 1 year.

**Results:**

PTH occurred in 20 (21.05%) out of a series of 95 patients who sustained a TBI for 1 year, due to CSF disturbance, triggering ventricular enlargement, and presenting with intracranial hypertension. Acute subdural hematoma was found in 14 (70.0%) patients, while midline shift/cisternal compression was present in 13 (65.0%) patients. Both parameters were strongly correlated with increased ICP, which was associated with low admission Glasgow Coma Score (GCS), severe TBI, and an unfavorable outcome. Older age was also correlated with aSDH and severe TBI, and with a bad outcome when ICP was elevated.

**Conclusion:**

Low admission GCS, aSDH, brain edema (midline shifting/basal cisternal compression), elevated ICP, and older age are strong predictors of unfavorable outcome and considerably worse prognosis in TBI patients who develop PTH. It seems that only PTH patients with a syndrome of increased intracranial pressure (ICP) require unconditional surgery, while those with chronic symptoms may be observed for PTH progression.

## Introduction

Post-traumatic hydrocephalus (PTH) is a common but dangerous complication of a traumatic brain injury (TBI) (1–5), which may increase its morbidity and mortality and complicate the outcome (6). It is characterized by the progressive accumulation of cerebrospinal fluid (CSF) within the cranial compartment, with ventriculomegaly secondary to traumatic deviations that disrupt CSF circulation and absorption. Its etiology is complex and multifactorial, including severe TBI, epidural hematoma (EDH), post-traumatic subarachnoid hemorrhage (SAH), acute subdural hematoma (aSDH), intraventricular hemorrhage (IVH), and decompressive craniectomy (DC). Yet, the exact mechanisms of PTH development, including CSF disturbances, supported by neuroinflammation and subarachnoid obstruction, remain inadequately determined. However, the PTH clinical presentation remains largely atypical. Consequently, diagnosing and managing PTH patients is demanding and logistically challenging.

The main aspects of PTH investigation in this narrative review are aimed at highlighting its incidence and the specific mechanisms of its development, as well as identifying predictors of PTH progression and the effectiveness of management strategies.

### Incidence

Since it is mainly grounded on different causes and various diagnostic criteria, PTH incidence in adults may differ substantially, influencing TBI morbidity and mortality if it is not recognized and treated early enough (7–10). Therefore, its incidence varies widely due to its predominantly atypical presentation and the disparity in diagnostic criteria (8, 9, 11–13). Recent studies of surgically treated patients suffering from PTH found that the overall incidence was 0.15 and 1.1% among those with moderate to severe TBI (14), while the overall incidence of PTH patients requiring shunt surgery was 0.94% (15). Yet, some authors suggest that a substantial amount of TBI survivors may develop post-traumatic ventriculomegaly weeks to months after brain injury (16), especially those who underwent DC, which is a well-known risk for PTH development (17, 18), affecting 11.9%–36% of patients undergoing this procedure (10, 19). A systematic review and meta-analysis found a statistically significantly higher occurrence of PTH, which was identified in patients undergoing DC for TBI compared to those that were managed without DC (20). In contrast, the peak time of PTH occurrence is noted during the first 3 months post brain injury (21).

### Diagnostics and clinical presentation

The most appropriate diagnostic approach for patients with PTH involves a combination of evolving clinical conditions and neuroradiological imaging (22), although there is no gold standard for PTH detection at the moment (23).

Typical clinical presentation in PTH adult patients is difficult to detect because the symptoms are usually masked by the underlying consequences of TBI. Therefore, the diagnosis of PTH is complex due to unresolved or additional effects of the primary or secondary TBI. A lack of clinical and functional improvement, which is inconsistent with the severity of the injury during the rehabilitation, is an early sign of PTH (24).

Diagnosing PTH in unconscious TBI patients is additionally challenging. Hence, it is sometimes difficult to differentiate between PTH and compensatory ventricular enlargement caused by secondary cortical atrophy following parenchymal damage to brain tissue (24). Therefore, this TBI complication should be closely monitored from the acute stage, especially in elderly patients (25). Potential risk factors for PTH include various types of coexisting intracranial hemorrhages evident on brain computed tomography (CT) scans (10). A high incidence of subdural hygroma after DC that may precede PTH was also reported (10, 17, 26).

Progressive ventricular enlargement revealed on repeated non-contrast brain CT scans is the essential diagnostic sign of PTH (8). The increased Evans’ index (EI – the ratio of maximal width of the frontal horns and maximal width of the inner skull) (27), and modified frontal horn index (FHI – the distance between the tips of the frontal horns) greater than 33% (28), as well as the presence of Gudeman CT scan criteria (lateral and third ventricular enlargement, and periventricular effusions) (29), are important volumetric parameters indicating PTH (30).

Measurements obtained through brain magnetic resonance imaging (MRI) provide a precise TBI evaluation and are also valuable for post-traumatic headache and PTH diagnostics (31).

Post-traumatic hydrocephalus may develop gradually within 2 weeks to a couple of years after the injury. It commonly occurs during the post-acute to late phase of a severe TBI (9), with the most likely diagnostic timeframe being within the first 50 days (32). Interhemispheric subdural hygromas and younger age are associated with shunt-dependent hydrocephalus, which appears to increase after DC in patients with severe TBI (10, 33).

The diagnostic criteria to distinguish PTH from post-traumatic ventriculomegaly (PTV) or cerebral atrophy include a different clinical course, imaging features, and CSF dynamics.

A sudden or gradual neurological decline after an initial stabilization is a key clinical characteristic of PTH, while a stable neurologic deficit corresponding to a pre-existing lesion is typical for PTV.

Progressive ventricular enlargement, particularly temporal horns, with periventricular trans-ependymal effusions, is a key radiological sign of PTH, while stable ventricular enlargement without periventricular edema related to brain tissue lost is typical for PTV.

Considering CSF dynamics, PTH is often accompanied by increased ICP, while its values in PTV remain normal.

### Management strategies

Ventriculoperitoneal (VP) shunting is a common treatment option, leading to a usually favorable outcome (34). However, it seems that only patients with PTH and elevated intracranial pressure (ICP) require unconditional VP shunting as the most common treatment option.

After TBI, external ventricular drainage (EVD) may be used to monitor ICP and reduce it if elevated, as well as to drain CSF. This procedure can also help prevent and treat PTH by facilitating CSF resorption, potentially decreasing the need for VP shunting (35).

The use of endoscopic third-ventriculostomy (ETV) for the treatment of PTH remains controversial (36). Nevertheless, an endoscope offers great management potential since it is more cost-effective, requires less time, and poses a lower risk of infection (37).

Earlier VP shunting is often associated with improved rehabilitation outcomes in most PHT cases (19, 38–40), regardless of gender and age, which appear to have no major influence on the outcome (39, 41). Nonetheless, it seems that only patients with PTH and intracranial hypertension require unconditional surgery, while those with chronic symptoms may only be observed for PTH progression (12). This is consistent with the findings on a cohort of PTH adult patients from our series.

This narrative review aims to discuss the mechanisms of PTH development, predictors of its progression, and the effectiveness of its surgical management in adult TBI patients. It seeks to comprehend recent literature on this clinical argument. It is also based on a single-center retrospective analysis of a cohort of adult PTH patients treated over 1 year.

## Materials and methods

All TBI adult patients who were operated on for 1 year, and who fulfilled the radiological PTH criteria based on ventricular size changes, were included in the study.

A series of retrospectively collected data was analyzed, including gender and age, the severity of TBI assessed by the admission Glasgow Coma Scale (GCS), and the presence of EDH, post-traumatic SAH, aSDH, IVH, and/or subdural hygroma visualized by CT brain scanning.

All patients underwent temporary EVD, and ICP was monitored in all of them. The ICP measurements were obtained immediately after successful EVD placement.

The elevation of ICP was the main diagnostic criterion in our case series, representing the rationale for dividing patients into case and control groups.

Utilizing an ICP threshold of ICP > 25 mmHg was the main rationale to explore the correlation between elevated ICP and the risk of PTH development.

All PTH patients were divided into two groups based on changes in ICP values. The case group was formed from patients having elevated ICP (ICP > 24 mmHg), while patients with normal ICP values were allocated to the control group. Both groups were regularly clinically and radiologically followed up during and after hospital discharge for 1 year.

Post-traumatic changes in ventricular size were measured on post-operative and check-up brain CT scans using PTH criteria, while the extent of ventricular enlargement was quantified by the EI and FHI.

The outcome was assessed by Glasgow Outcome Scale Extended (GOS-E) at hospital discharge and at regular follow-ups, up to one-year post-injury.

The TBI patients having previous cranial surgeries and/or cranial irradiation, as well as those on antiplatelet/anticoagulant therapy, were excluded from the analysis.

Insights into the topic’s current evidence were provided employing a narrative review that presents a summation of available literature, which was identified by searching academic databases using keywords related to the topic, such as: post-traumatic hydrocephalus; mechanisms; progression predictors; and management strategies.

### Statistical analysis

Statistical Package for the Social Sciences (SPSS) for Windows Version 26 (IBM Deutschland GmbH, Ehningen, Germany) was used to process, analyze, and assess clinical and radiological PHT characteristics, volumetric measurements, and surgical techniques in these case series patients.

Mean Standard Deviation was used for reporting normal vs. pathological ranges of clinical and radiological PHT characteristics. Pearson correlation was employed for determining the strength of the relationship between continuous variables gained by volumetric measurements. Longitudinal analysis was employed for the statistical assessment of surgical techniques.

The conventional level of statistical significance (*p* < 0.05) was employed.

The *Phi* correlation coefficient (equivalent to the Pearson correlation coefficient when two binary variables are observed) has been used to measure the association between the clinical scores in the case group and the control group.

## Results

This retrospective cohort analysis included 95 brain-injured patients who were admitted to the Department of Neurosurgery, University Hospital Center Sestre milosrdnice, Zagreb, Croatia, between January and the end of December 2021 to treat the consequences of severe TBI. Among them, 20 (21.05%) patients developed ventricular enlargement and PTH, despite being early treated by the EVD.

Male predominance (14/20; 70.0%) was observed in the cohort of PTH patients. The youngest patient was a 20-year-old male, while the oldest one was an 85-year-old male. The mean age of the entire group was 59.4 years.

The PTH patients were divided into the case and control groups. The case group was comprised of 15 (75.0%) patients with an increased ICP (>25 mm Hg) who suffered a severe TBI (GCS = 3–8), while the remaining 5 (25.0%) patients with normal ICP values who sustained mild to moderate TBI were allocated to the control group.

**Figure 1:**
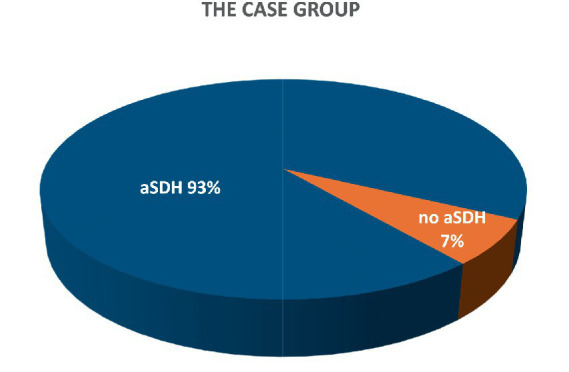
Distribution of aSDH in the case group patients (increased ICP).

Acute subdural hematoma was found in 14 (70.0%) out of 20 patients, i.e., in 14 out of 15 (93%) patients in the case group and in none of the patients in the control group (Figure 1). A strong positive correlation (*Phi* correlation coefficient = 0.88) existed between acute subdural hematoma and increased ICP (Figure 1).

**Figure 2:**
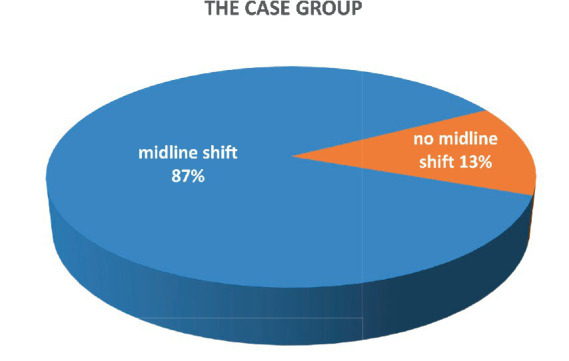
Distribution of patients from the case group (increased ICP) with midline shift and/or basal cistern compression.

Midline shift and/or basal cistern compression were associated with PTH in 13 (65.0%) patients, i.e., in 13 out of 15 (87%) in the case group (Figure 2) and in none of the patients in the control group. A strong positive correlation (*Phi* correlation coefficient = 0.79) existed between midline shift and/or basal cistern compression and increased ICP.

**Figure 3:**
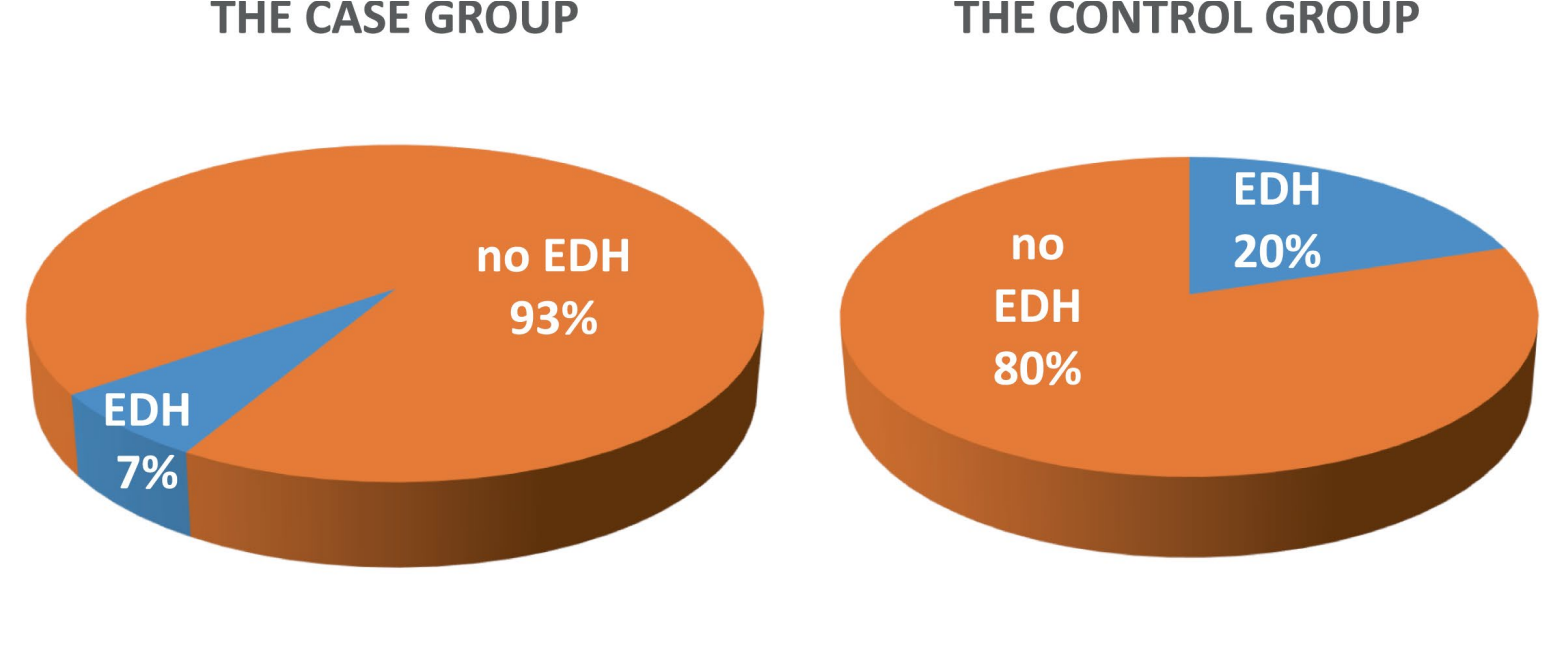
Distribution of patients with EDH in the case group (increased ICP) – left; and in the control group (normal ICP) – right.

Epidural hematoma was present in 2 (10.0%) of 20 PTH patients, i.e., in 1 out of 15 (6.7%) in the case group and in 1 out of 5 patients (20%) in the control group (Figure 3). A very weak negative correlation (*Phi* correlation coefficient = − 0.19) existed between EDH and increased ICP.

**Figure 4:**
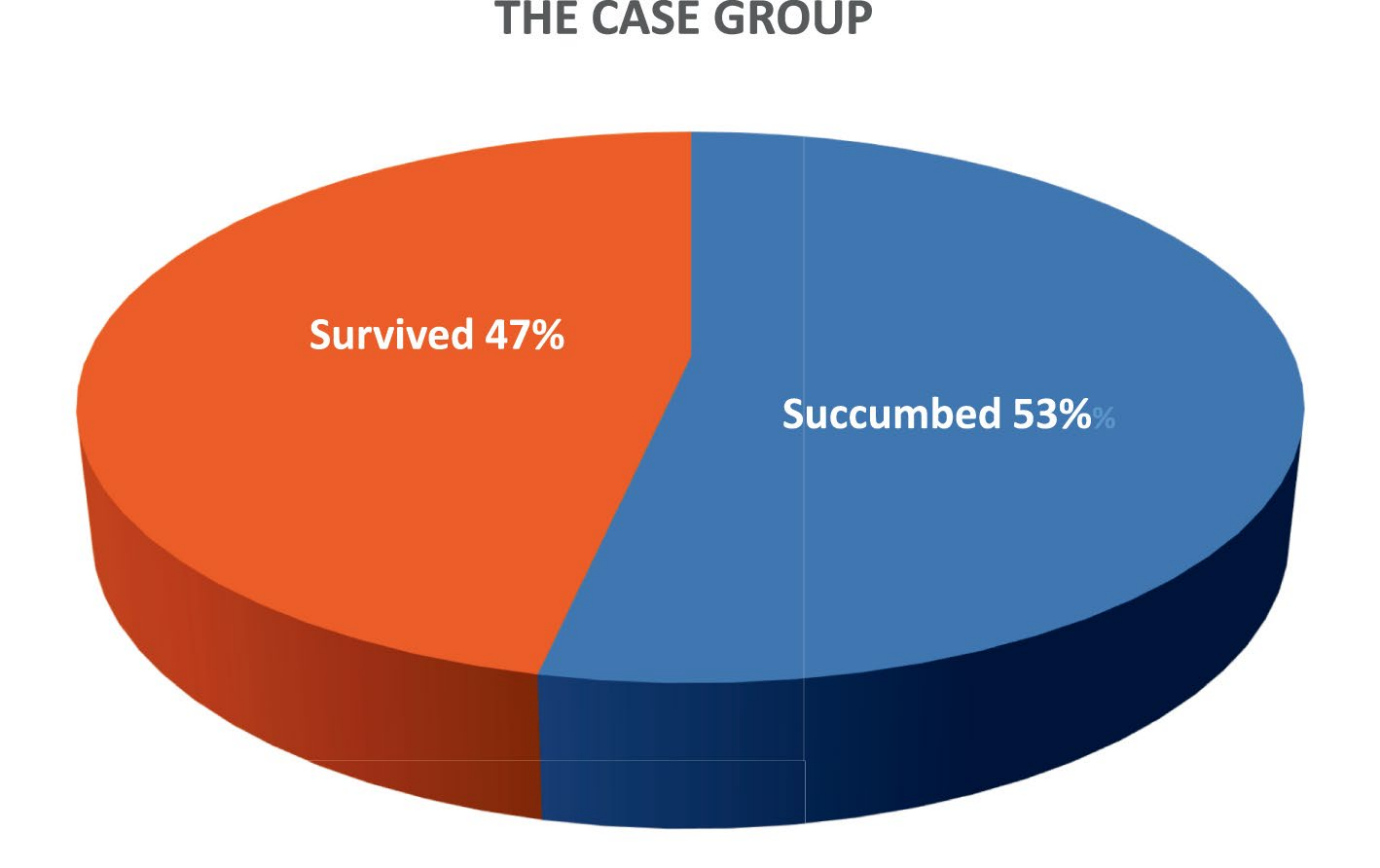
Distribution of patients with increased ICP who had survived or had a severe TBI.

Eight (40.0%) out of 20 PTH patients died from severe TBI, while 12 (60.0%) survived. Specifically, 8 out of 15 (53%) patients in the case group succumbed to severe TBI (Figure 4), and no patients in the control group did. A moderate positive correlation (*Phi* correlation coefficient = 0.47) existed between increased ICP and severe TBI.

**Figure 5:**
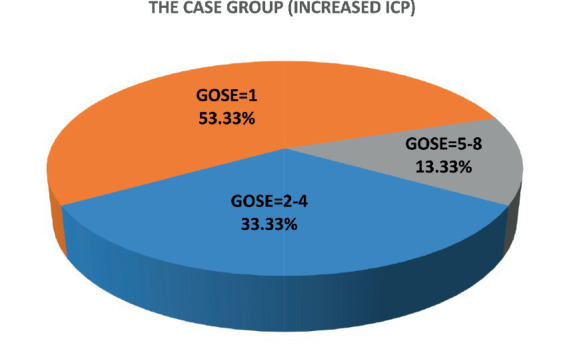
Outcome distribution (GOS-E) of case group patients with increased ICP.

A strong negative correlation (*Phi* correlation coefficient = − 0.78) existed between increased ICP and full or moderate patient recovery, and a weak positive correlation exists between increased ICP and unfavorable outcome (*Phi* correlation coefficient = 0.33), as 5 out of 15 patients (33.33%) with increased ICP had an unfavorable outcome (Figure 5).

**Figure 6:**
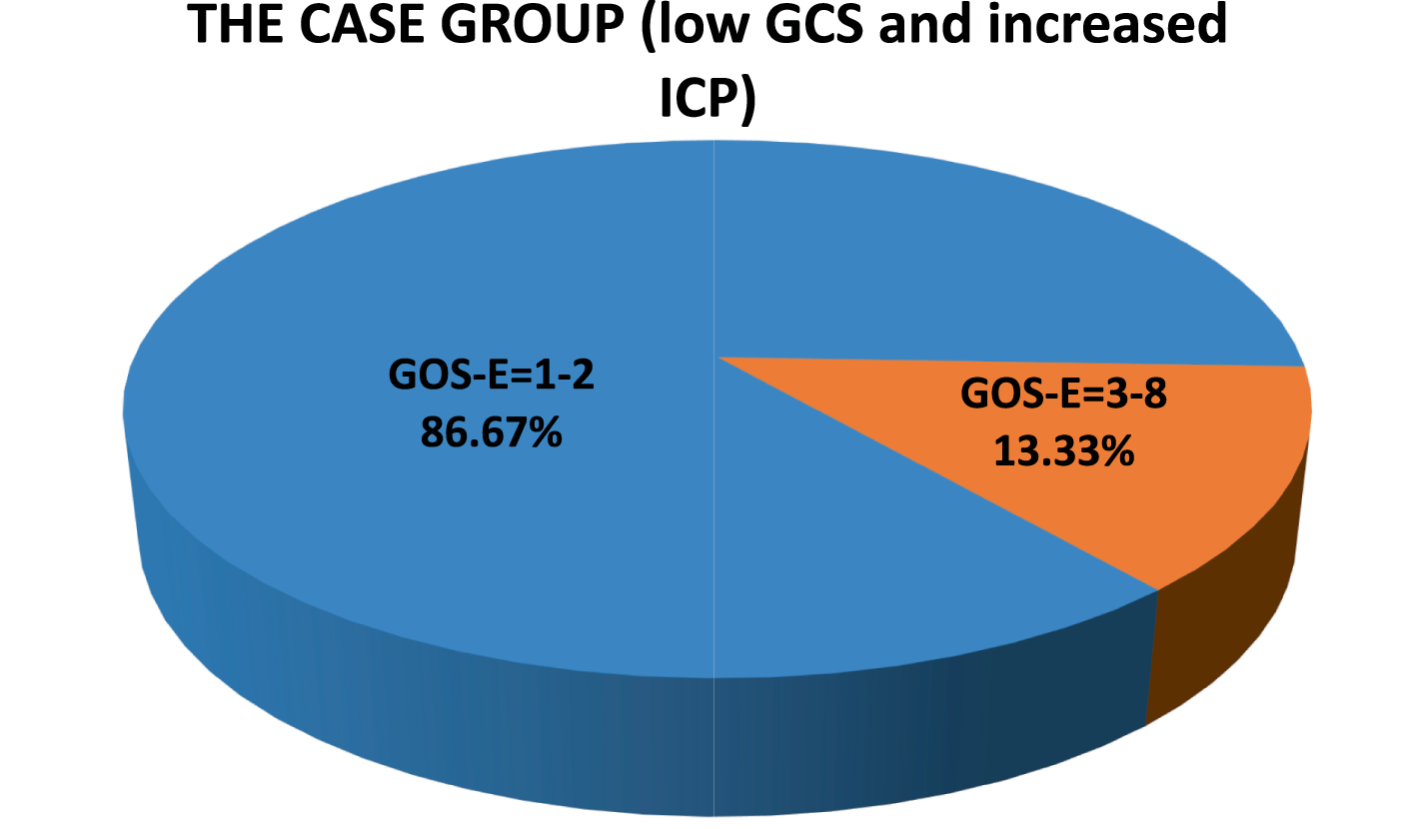
Outcome distribution (GOS-E) of case group patients with low GCS and increased ICP.

All patients with increased ICP had low admission GCS (GCS = 3–8), and 13 out of 15 (86.67%) either succumbed to severe TBI or had an unfavorable outcome (GOS-E 1–2) (Figure 6). In contrast, none of the patients in the control group had a low admission GCS or an unfavorable outcome. The outcome in the control group was enhanced in comparison to the case group.

**Figure 7:**
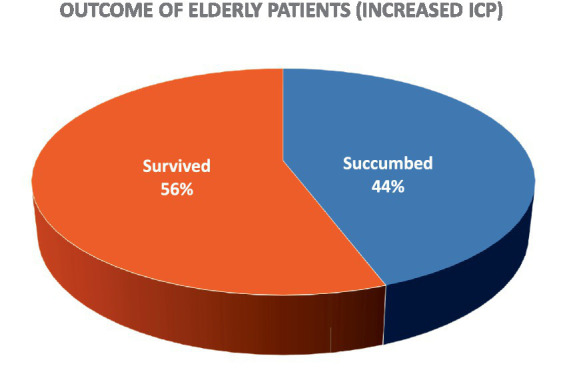
Outcome distribution (GOS-E) in elderly patients with increased ICP.

Four out of 9 (44.44%) elderly patients (older than 65 years) in the case group and none of the two elderly patients in the control group experienced an unfavorable outcome. A weak positive correlation (*Phi* correlation coefficient = 0.36) was observed between older age and an unfavorable outcome when ICP was elevated. Four out of 9 (44.44%) elderly patients in the case group (Figure 7) and none of the 2 elderly patients in the control group succumbed to severe TBI.

All elderly patients from the case group (9 out of 9; 100%) and no elderly patients from the control group (0 out of 2) experienced aSDH. A very strong positive correlation (*Phi* correlation coefficient = 1) existed between older age and aSDH when ICP was increased.

**Figure 8:**
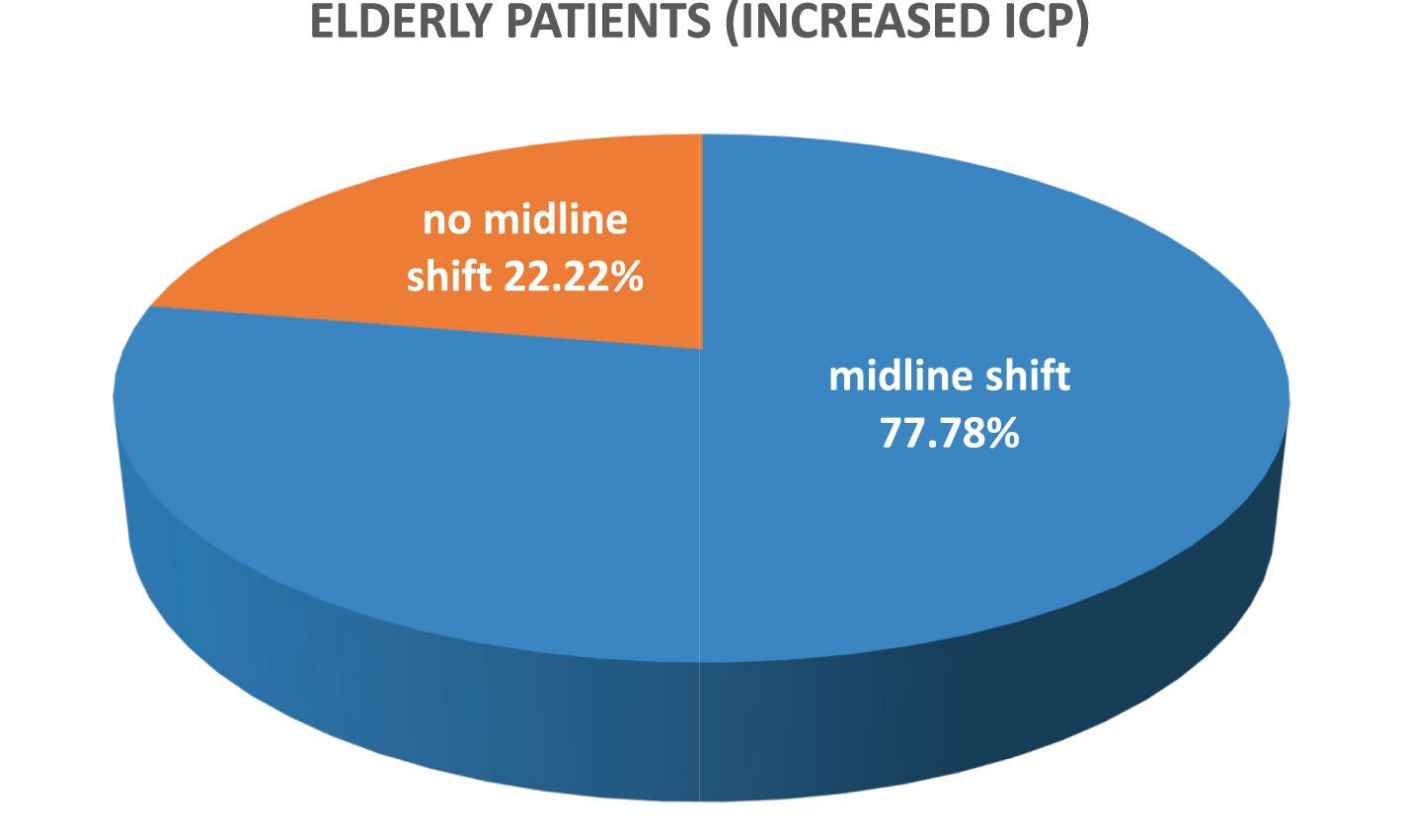
Distribution of elderly patients with increased ICP according to midline shift/basal cistern compression.

Seven out of 9 (77.78%) elderly patients with increased ICP (Figure 8), and none of the two elderly patients in the control group had midline shift/basal cistern compression. A moderate to strong positive correlation (*Phi* correlation coefficient = 0.62) existed between older age and midline shift/basal cistern compression when ICP was increased.

## Discussion

The classification of hydrocephalus as either non-communicating or communicating was first introduced by Dandy and Blackfan in 1914 (42). In non-communicating (obstructive) hydrocephalus, normal CSF flow is obstructed, leading to ventricular dilatation and mass effect due to elevated ICP. On the contrary, severe TBI accompanied by subarachnoid/intracranial/intraventricular hemorrhage, and/or meningitis may also produce communicating (external) PTH (7), where full CSF flow between the ventricles and subarachnoid space exists, and ICP is not increased, but CSF absorption remains severed, resulting in excessive accumulation of CSF in the subarachnoid space at the brain convexity. Eventually, any PTH occurs due to a disproportion in CSF flow, triggering ventricular enlargement and presenting as a syndrome of increased ICP^12^. Medically refractory intracranial hypertension can be induced by many intracranial pathologies, including secondary brain damage in severely injured patients (43–45). That was the reason why all the PTH patients from our series were treated by EVD.

Severe IVH after TBI promotes the formation of PTH and abnormal CSF flow through an obstructive mechanism (46). It damages the brain metabolism and reduces clinical improvement, causing neurological deterioration and poorer outcomes if not detected and treated soon enough (47).

The PTH diagnosis is generally based on clinical presentation and radiological findings, although the applied diagnostic criteria for PTH differ between studies (17). At present, there is no universally accepted diagnostic standard for its detection (23). That is why the percentage of patients experiencing PTH in trauma cohorts varies greatly, ranging from 0.7% to 51.4% (14). It was 20.05% of our series. The incidence in our series was much higher than the one previously reported in the literature, but was more similar to the 17% incidence rate of PTH reported by Chibbaro et al. (48).

In adults, PTH is typically diagnosed between 3 and 12 months after a brain injury, usually during rehabilitation, and is therefore known as secondary hydrocephalus.

Symptoms may include urinary incontinence, walking difficulties, cognitive impairment, headache, nausea, and vomiting, increased ICP, particularly if the condition that causes it is acute. Sometimes, PTH may be clinically similar to normal pressure hydrocephalus (NPH) and present as Hakim’s triad (gait deterioration, cognitive decline, and urinary incontinence) (6).

Managing and caring for PTH patients is rather demanding and logistically challenging. It involves meticulous diagnostics, EVD or V-P shunting, frequent follow-ups, regular neuroimaging, and treating complications such as CSF leak, shunt malfunction, obstruction, or over-drainage, as well as infections.

**Figure 9:**
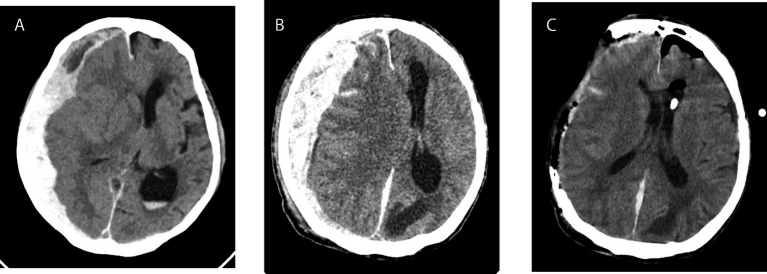
Initial axial brain CT scan showing acute subdural hematoma (aSDH) of the right cerebral hemisphere, compression of the right lateral and third ventricle, subfalcine and uncal herniation, and midline shift to the left for 1.7 cm. A considerable amount of blood is seen in the enlarged temporal horn of the left lateral ventricle **(A)**. Initial native axial brain CT scan of another patient showing a large peri-hemispheric right-sided SDH with compressive mass effect and a substantial midline shift, displacing the supratentorial ventricles contralaterally. Initial ventricular dilatation with enlargement of the left lateral ventricle’s temporal horn is also visible **(B)**. Post-operative CT scan of the same patient depicting resolution of aSDH and mass effect, slight ventriculomegaly, and the tip of the EDV placed in the left lateral ventricle frontal horn **(C)**.

Identifying predictors of PTH development and progression, including low GCS, EDH, post-traumatic SAH, aSDH, IVH, and DC, highlights the importance of close monitoring of TBI patients (49). A clear association between injury severity and PTH has been established (50), suggesting that the extent of primary brain injury may be more responsible for the damage to CSF pathways, leading to the development of PTH. The acute subdural hematoma, as a variable most strongly associated with PTH, was present in a significant majority of our patients, which is consistent with recent literature (41). A strong positive correlation (*Phi* correlation coefficient = 0.88) existed between aSDH and increased ICP (Figure 1). Peri-hemispheric aSDH causing ventricular compression and dislocation, subfalcine and uncal herniation, and midline shift represents a clear surgical indication (Figures 9A–C).

**Figure 10:**
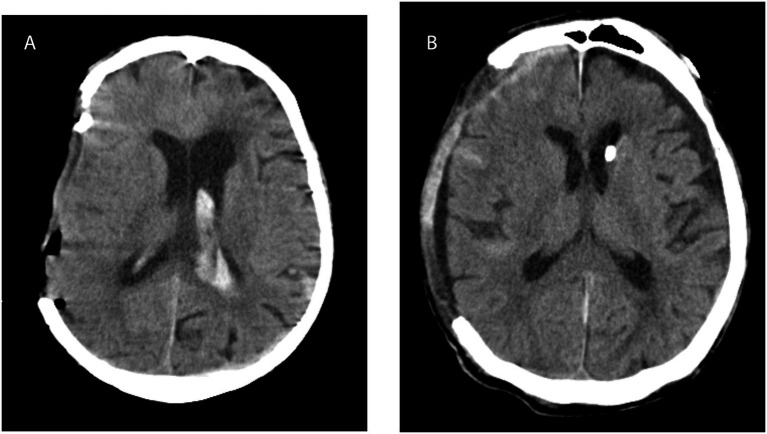
Control axial brain CT performed on day 5 post-injury, illustrating a substantial decrease in midline shifting after a right-sided decompressive craniectomy and evacuation of aSDH, as well as the development of PTH together with the progression of intraventricular hemorrhage **(A)**. Control axial brain CT of another patient at 3 weeks post-procedure, revealing no midline shift, a PTH with lesser ventricular enlargement, and regressive dynamics of hemorrhagic focal lesions **(B)**.

Other PTH-associated variables may include midline shifting/basal cisterns compression, advanced age, subarachnoid/intracranial/intraventricular hemorrhage, subdural hygroma, duraplasity (11), and CSF infection. According to our findings, a strong positive correlation (*Phi* correlation coefficient = 0.79) existed between midline shift and/or basal cistern compression and increased ICP (Figure 2). Additionally, a strong positive correlation (*Phi* correlation coefficient = 0.79) was observed between midline shift and/or basal cistern compression and increased ICP (Figure 2). However, a considerable decrease in midline shifting after surgical evacuation of aSDH may be accompanied by the development of PTH, together with the progression of intraventricular hemorrhage, which was the case with some patients from our series (Figures 10A,B).

At the same time, a very weak negative correlation (*Phi* correlation coefficient = − 0.19) existed between increased ICP and EDH (Figure 3), suggesting that EDH *per se* may not be associated with intracranial hypertension, which is mostly caused by a mass (compressive) effect that an EDH produces. However, we observed a moderate positive correlation (*Phi* correlation coefficient = 0.47) between increased ICP and severe TBI (Figure 4), indicating that the severity of injury is usually accompanied and directly associated with a rise in ICP values.

**Figure 11:**
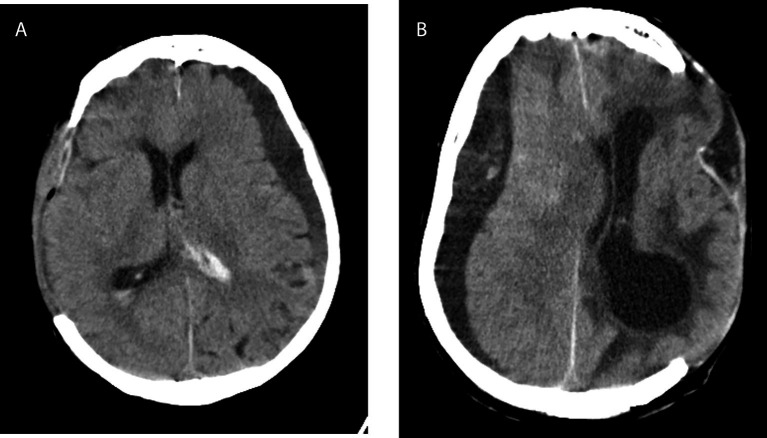
Check-up axial brain CT scan on day 9 post-injury showing a regressive dynamic of intracranial hemorrhage and brain edema, but also the contralateral formation of subdural hygroma producing a midline shift to the right for 7 mm **(A)**. Check-up axial brain CT of another patient on day 15 post-injury, depicting a peri-hemispheric right-sided subdural hygroma with a mass effect and midline shift, contralateral to the evacuated left-sided aSDH and accompanied by the right lateral ventricle compression, as well as dilatation and displacement of the left lateral ventricle, whose temporal horn is extremely enlarged **(B)**.

Further post-surgical turbulence in CSF dynamics was associated with contralateral formation of subdural hygroma (Figures 11A,B) and unfavorable long-term outcome (51).

According to our findings, key predictors of PTH progression included different types of intracranial hemorrhage causing mass effect, midline shift, and basal cisternal compression, as well as advanced age at surgery. On the contrary, according to a recent systematic review and meta-analysis, age was not significantly correlated with PTH (49).

In our series, a strong negative correlation (*Phi* correlation coefficient = − 0.78) existed between increased ICP and a favorable outcome. It means that the ICP values remained within normal range in TBI patients having moderate to full recovery. Simultaneously, a weak positive correlation existed between increased ICP and unfavorable outcome (*Phi* correlation coefficient = 0.33) (Figure 5), confirming increased ICP as a predictor of bad prognosis. All patients with increased ICP had low admission GCS (GCS = 3–8), and 13 out of 15 (86.67%) either succumbed to severe TBI or had an unfavorable outcome (GOS-E 1-2) (Figure 6). In contrast, none of the patients in the control group had a low admission GCS or an unfavorable outcome. The outcome in the control group was enhanced in comparison to the case group, confirming that elevated ICP was a strong predictor of poorer prognosis.

According to the relevant literature, low levels of consciousness (admission GCS < 8), IVH, as well as contralateral subdural hygroma, are identified as independent risk factors for PTH development (52), which mostly occurs gradually, during the rehabilitation of severely injured patients (45, 52, 53). Low GCS and contralateral subdural hygroma were also identified as strong predictors of PTH progression and worse outcome in our series. Recent findings suggest that post-traumatic SAH, basal cisterns compression, and contusion hemorrhage progression, as well as advanced age, are independent risk factors for PTH development and progression (54).

In our series, a weak positive correlation (*Phi* correlation coefficient = 0.36) was observed between older age and an unfavorable outcome when ICP was elevated. A weak positive correlation (*Phi* correlation coefficient = 0.36) existed between older age and severe TBI when ICP was increased (Figure 7). A very strong positive correlation (*Phi* correlation coefficient = 1) existed between older age and aSDH when ICP was increased. Simultaneously, a moderate to strong positive correlation (*Phi* correlation coefficient = 0.62) existed between older age and midline shift/basal cistern compression when ICP was increased (Figure 8).

The above parameters were strong predictors of PTH and unfavorable outcomes.

Comprehending the literature, the majority of PTH patients have been treated with permanent CSF derivation by VP shunts (2, 39, 55). Lang et al. (56) demonstrated that decompressive craniectomy for severe TBI is the triggering factor causing fundamental derangement of CSF dynamics, which explains why many TBI patients require urgent VP shunt, as well as cranioplasty to prevent the development of PTH. However, early EDV is essential for PTH prevention and progression. That was the reason why all patients from our series underwent temporary EVD, and ICP was monitored in all of them.

### Closing remarks

This paper aims to enhance clinical decision-making that improves patient outcomes by investigating PTH development, predictors of its progression, and the relationship between surgical management strategies and outcomes.

Post-traumatic hydrocephalus is a specific type of ventricular dilatation that occurs as a serious complication of moderate to severe TBI, which may increase TBI morbidity and mortality, as well as compromise the outcomes.

Assessing the efficacy of PTH management and predicting its progression is difficult due to the heterogeneity of TBI severity, PTH variability, varying diagnostic criteria, and diverse surgical treatment methods.

Low admission GCS, aSDH, midline shift/cisternal compression, and elevated ICP in TBI patients who developed PTH are strong indicators of an unfavorable outcome and predictors of a considerably worse prognosis—the results from our series support all of these.

One must always take into consideration the development of PTH in days/weeks/months following a moderate to severe TBI. Early EDV is a prerequisite for PTH prevention, whereas VP shunting is the preferred surgical management for PTH.

### Strengths and limitations

The strength of the manuscript arises from its combination of a narrative review with a single institution’s series of clinical data.

The paper’s limitations originate from the small sample size in the investigated cohort and its single-center retrospective character, which may reduce the generalizability of the findings.

Considering this, our findings should be assumed as rather observational than definitive.

## Conclusion

Assessing the mechanisms and efficacy of PTH surgical management is challenging due to the heterogeneity of TBI severity, PTH variations, and uncertain diagnostic criteria, as well as disparities in the length of time needed for the development of PTH following a TBI.

Low admission GCS, aSDH, brain edema (midline shifting/basal cisternal compression), and elevated ICP in TBI patients who develop PTH are strong predictors of unfavorable outcome and considerably worse prognosis. Acute SDH/hygroma is the variable mostly associated with PTH, and it is recorded in a substantial majority of patients. Other variables included: advanced age, post-traumatic intracranial hemorrhage, and mass effect contributing to intracranial hypertension.

Early EVD is a prerequisite for PTH prevention, while VP shunting is a preferable method of PTH surgical management in adults.

## Data Availability

The original contributions presented in the study are included in the article/supplementary material, further inquiries can be directed to the corresponding author.

## References

[1] Bontke CF. Medical complications related to traumatic brain injury. Phys Med Rehabil: State Art Rev. (1989) 3:43–58.

[2] Kim H Lee HS Ahn SY Park SC Huh W. Factors associated with postoperative hydrocephalus in patients with traumatic acute subdural hemorrhage. J Korean Neurosurg Soc. (2017) 60:730–7. doi: 10.3340/jkns.2017.0210, 29142634 PMC5678061

[3] Narayan RJ Gokaslan ZL Bontke CF. "Neurologic sequelae of head injury". In: Rosenthal M, editor. Rehabilitation of the Adult and Child with Traumatic Brain Injury. Philadelphia, PA: DAVIS Construction (1990)

[4] Oberholzer M Muri RM. Neurorehabilitation of traumatic brain injury (TBI): a clinical review. Med Sci (Basel). (2019) 7:47. doi: 10.3390/medsci7030047, 30889900 PMC6473767

[5] Stein S Schrader P. Neurologic sequelae. Phys Med Rehabil: State Art Rev. (1990) 4:543–57.

[6] Sanker V Kundu M El Kassem S El Nouiri A Emara M Maaz ZA . Posttraumatic hydrocephalus: recent advances and new therapeutic strategies. Health Sci Rep. (2023) 6:e1713. doi: 10.1002/hsr2.1713, 38028696 PMC10652704

[7] Adams RD Victor M. "Disturbances of cerebrospinal fluid and its circulation, including hydrocephalus and meningeal reactions". In: Principles of Neurology. New York, NY: McGraw-Hill Information Services Co (1989)

[8] Groswasser Z Cohen M Reider-Groswasser I Stern MJ. Incidence, CT findings and rehabilitation outcome of patients with communicative hydrocephalus following severe head injury. Brain Inj. (1988) 2:267–72. doi: 10.3109/02699058809150897, 3203174

[9] Mazzini L Campini R Angelino E Rognone F Pastore I Oliveri G. Posttraumatic hydrocephalus: a clinical, neuroradiologic, and neuropsychologic assessment of long-term outcome. Arch Phys Med Rehabil. (2003) 84:1637–41. doi: 10.1053/s0003-9993(03)00314-9 14639563

[10] Vedantam A Yamal JM Hwang H Robertson CS Gopinath SP. Factors associated with shunt-dependent hydrocephalus after decompressive craniectomy for traumatic brain injury. J Neurosurg. (2018) 128:1547–52. doi: 10.3171/2017.1.JNS162721, 28621627

[11] Ding J Guo Y Tian H. The influence of decompressive craniectomy on the development of hydrocephalus: a review. Arq Neuropsiquiatr. (2014) 72:715–20. doi: 10.1590/0004-282x20140106, 25252237

[12] Guyot LL Michael DB. Post-traumatic hydrocephalus. Neurol Res. (2000) 22:25–8. doi: 10.1080/01616412.2000.11741034, 10672577

[13] Mori K Shimada J Kurisaka M Sato K Watanabe K. Classification of hydrocephalus and outcome of treatment. Brain Dev. (1995) 17:338–48. doi: 10.1016/0387-7604(95)00070-r, 8579221

[14] Heinonen A Rauhala M Isokuortti H Kataja A Nikula M Öhman J . Incidence of surgically treated post-traumatic hydrocephalus 6 months following head injury in patients undergoing acute head computed tomography. Acta Neurochir. (2022) 164:2357–65. doi: 10.1007/s00701-022-05299-3, 35796788 PMC9427877

[15] Pan J Feroze AH McGrath M Eaton J Abecassis IJ Temkin N . Incidence and risk model of post-traumatic hydrocephalus in patients with traumatic brain injury. World Neurosurg. (2024) 185:e491–9. doi: 10.1016/j.wneu.2024.02.060, 38369109

[16] Marmarou A Foda MA Bandoh K Yoshihara M Yamamoto T Tsuji O . Posttraumatic ventriculomegaly: hydrocephalus or atrophy? A new approach for diagnosis using CSF dynamics. J Neurosurg. (1996) 85:1026–35. doi: 10.3171/jns.1996.85.6.1026 8929491

[17] De Bonis P Sturiale CL Anile C Gaudino S Mangiola A Martucci M . Decompressive craniectomy, interhemispheric hygroma and hydrocephalus: A timeline of events? Clin Neurol Neurosurg. (2013) 115:1308–12. doi: 10.1016/j.clineuro.2012.12.011, 23290122

[18] Fattahian R Bagheri SR Sadeghi M. Development of posttraumatic hydrocephalus requiring ventriculoperitoneal shunt after decompressive craniectomy for traumatic brain injury: a systematic review and meta-analysis of retrospective studies. Med Arch. (2018) 72:214–9. doi: 10.5455/medarh.2018.72.214-9, 30061770 PMC6021151

[19] Goldschmidt E Deng H Puccio AM Okonkwo DO. Post-traumatic hydrocephalus following decompressive hemicraniectomy: incidence and risk factors in a prospective cohort of severe TBI patients. J Clin Neurosci. (2020) 73:85–8. doi: 10.1016/j.jocn.2020.01.027, 31987632

[20] Mavrovounis G Kalogeras A Brotis A Iaccarino C Demetriades AK Fountas KN. Incidence of post-traumatic hydrocephalus in traumatic brain injury patients that underwent DC versus those that were managed without DC: a systematic review and meta-analysis. Brain Spine. (2021) 1:100303. doi: 10.1016/j.bas.2021 36247396 PMC9560681

[21] Chen KH Lee CP Yang YH Yang YH Chen CM Lu ML . Incidence of hydrocephalus in traumatic brain injury: a nationwide population-based cohort study. Medicine. (2019) 98:e17568. doi: 10.1097/MD.0000000000017568, 31626123 PMC6824727

[22] Iaccarino C Chibbaro S Sauvigny T Timofeev I Zaed I Franchetti S . Consensus-based recommendations for diagnosis and surgical management of cranioplasty and post-traumatic hydrocephalus from a European panel. Brain Spine. (2024) 4:102761. doi: 10.1016/j.bas.2024.102761, 38510640 PMC10951750

[23] Cava FC Castellani GB Maietti E Salucci P Colombo V Palandri G. A new clinical protocol for a timely diagnosis and treatment of hydrocephalus in patients with severe acquired brain injury. Brain Sci. (2023) 13:1067. doi: 10.3390/brainsci13071067, 37508999 PMC10377718

[24] Akira M Yuichi T Tomotaka U Takaaki K Kenichi M Chimi M. The outcome of neurorehabilitation efficacy and management of traumatic brain injury. Front Hum Neurosci. (2022) 16:870190. doi: 10.3389/fnhum.2022.870190, 35814948 PMC9256961

[25] Kammersgaard LP Linnemann M Tibæk M. Hydrocephalus following severe traumatic brain injury in adults. Incidence, timing, and clinical predictors during rehabilitation. NeuroRehabilitation. (2013) 33:473–80. doi: 10.3233/NRE-130980, 23949078

[26] Nasi D Gladi M Di Rienzo A di Somma L Moriconi E Iacoangeli M . Risk factors for post-traumatic hydrocephalus following decompressive craniectomy. Acta Neurochir. (2018) 160:1691–8. doi: 10.1007/s00701-018-3639-0, 30054725

[27] Evans JP Scheinker IM. Histologic studies of the brain following head trauma, II: post-traumatic petechial and massive intracerebral hemorrhage. J Neurosurg. (1946) 3:101–13. doi: 10.3171/jns.1946.3.2.0101 21018500

[28] Fotakopoulos G Tsianaka E Siasios G Vagkopoulos K Fountas K. Posttraumatic hydrocephalus after decompressive craniectomy in 126 patients with severe traumatic brain injury. J Neurol Surg A Cent Eur Neurosurg. (2016) 77:88–92. doi: 10.1055/s-0035-1558411, 26351868

[29] Gudeman SK Kishore PR Miller JD Girevendulis AK Lipper MH Becker DP. The genesis and significance of delayed traumatic intracerebral hematoma. Neurosurgery. (1979) 5:309–13. doi: 10.1227/00006123-197909000-00002, 503290

[30] Silva Neto AR Valença MM. Transcalvarial brain herniation volume as a predictor of posttraumatic hydrocephalus after decompressive craniectomy. Clin Neurol Neurosurg. (2019) 182:73–8. doi: 10.1016/j.clineuro.2019.05.003, 31096109

[31] Nasel C Gentzsch S Heimberger K. Diffusion-weighted magnetic resonance imaging of cerebrospinal fluid in patients with and without communicating hydrocephalus. Acta Radiol. (2007) 48:768–73. doi: 10.1080/02841850701408251, 17729009

[32] Hannah EM Zyck S Hazama A Krishnamurthy S. Scoping review of the risk factors and time frame for development of post-traumatic hydrocephalus. Rev Neurosci. (2021) 33:133–46. doi: 10.1515/revneuro-2021-0043, 34144640

[33] Lee SH Ko MJ Lee YS Cho J Park YS. Clinical impact of craniectomy on shunt-dependent hydrocephalus after intracerebral hemorrhage: a propensity score-matched analysis. Acta Neurochir. (2024) 166:34. doi: 10.1007/s00701-024-05911-8, 38270816

[34] Kim SW Lee SM Shin H. Clinical analysis of post-traumatic hydrocephalus. J Korean Neursurg Soc. (2005) 38:211–4.

[35] Chung DY Olson DM John S Mohamed W Kumar MA Thompson BB . Evidence-based management of external ventricular drains. Curr Neurol Neurosci Rep. (2019) 19:94. doi: 10.1007/s11910-019-1009-9, 31773310 PMC7383112

[36] De Bonis P Tamburrini G Mangiola A Pompucci A Mattogno PP Porso M . Post-traumatic hydrocephalus is a contraindication for endoscopic third-ventriculostomy: Isn’t it? Clin Neurol Neurosurg. (2013) 115:9–12. doi: 10.1016/j.clineuro.2012.08.021, 22925601

[37] Enslin J Thango NS Figaji A Fieggen GA. Hydrocephalus in low and middle-income countries - progress and challenges. Neurol India. (2021) 69:S292–7. doi: 10.4103/0028-3886.332285, 35102979

[38] Kowalski RG Weintraub AH Rubin BA Gerber DJ Olsen AJ. Impact of timing of ventriculoperitoneal shunt placement on outcome in posttraumatic hydrocephalus. J Neurosurg. (2018) 23:406–17. doi: 10.3171/2017.7.JNS17555 29473779

[39] Tribl G Oder W. Outcome after shunt implantation in severe head injury with post-traumatic hydrocephalus. Brain Inj. (2000) 14:345–54. doi: 10.1080/026990500120637 10815842

[40] Weintraub AH Gerber DJ Kowalski RG. Posttraumatic hydrocephalus as a confounding influence on brain injury rehabilitation: incidence, clinical characteristics, and outcomes. Arch Phys Med Rehabil. (2017) 98:312–9. doi: 10.1016/j.apmr.2016.08.478, 27670926

[41] Kim JH Ahn JH Oh JK Song JH Park SW Chang IB. Factors associated with the development and outcome of hydrocephalus after decompressive craniectomy for traumatic brain injury. Neurosurg Rev. (2021) 44:471–8. doi: 10.1007/s10143-019-01179-0, 31953782

[42] Dandy WE Blackfan KD. Internal hydrocephalus: an experimental, clinical, and pathological study. Am J Dis Child. (1914) 8:406:–482. doi: 10.1001/archpedi.1914.02180010416002

[43] Muzevic D Splavski B. The Lund concept for severe traumatic brain injury. Cochrane Database Syst Rev. (2013) 2013:CD010193–13. doi: 10.1002/14651858.CD010193.pub2, 24338524 PMC9986507

[44] Stiver SI. Complications of decompressive craniectomy for traumatic brain injury. Neurosurg Focus. (2009) 26:E7. doi: 10.3171/2009.4.FOCUS0965, 19485720

[45] Su TM Lan CM Lee TH Hsu SW Tsai NW Lu CH. Risk factors for the development of posttraumatic hydrocephalus after unilateral decompressive craniectomy in patients with traumatic brain injury. J Clin Neurosci. (2019) 63:62–7. doi: 10.1016/j.jocn.2019.02.006, 30827885

[46] Symss NP Oi S. Theories of cerebrospinal fluid dynamics and hydrocephalus: historical trend. J Neurosurg Pediatr. (2013) 11:170–7. doi: 10.3171/2012.3.PEDS0934, 23215851

[47] Chen H Yuan F Chen SW Guo Y Wang G Deng ZF . Predicting posttraumatic hydrocephalus: derivation and validation of a risk scoring system based on clinical characteristics. Metab Brain Dis. (2017) 32:1427–35. doi: 10.1007/s11011-017-0008-2, 28391551

[48] Chibbaro S Zaed I Dannhoff G Todeschi J Mallereau CH Prisco L . Cranioplasty complications in severe traumatic brain injury: implications of timing of surgery, implant material and incidence of ventriculomegaly versus post-traumatic hydrocephalus. Neurosurg Rev. (2025) 48:659. doi: 10.1007/s10143-025-03832-3, 40974389

[49] Xiao ZK Duan Y Gao G Wang Y Huang M Liu J . Risk factors for the development of hydrocephalus in traumatic brain injury: a systematic review and meta-analysis. Neurosurg Rev. (2025) 48:522. doi: 10.1007/s10143-025-03611-0, 40542903

[50] Honeybul S Ho KM. Incidence and risk factors for post-traumatic hydrocephalus following decompressive craniectomy for intractable intracranial hypertension and evacuation of mass lesions. J Neurotrauma. (2012) 29:1872–8. doi: 10.1089/neu.2012.2356, 22583391

[51] Di G Zhang Y Liu H Jiang X Liu Y Yang K . Postoperative complications influencing the long-term outcome of head-injured patients after decompressive craniectomy. Brain Behav. (2019) 9:e01179. doi: 10.1002/brb3.1179, 30511376 PMC6346640

[52] Lu VM Carlstrom LP Perry A Graffeo CS Domingo RA Young CC . Prognostic significance of subdural hygroma for post-traumatic hydrocephalus after decompressive craniectomy in the traumatic brain injury setting: a systematic review and meta-analysis. Neurosurg Rev. (2021) 44:129–38. doi: 10.1007/s10143-019-01223-z, 31845199

[53] Di G Hu Q Liu D Jiang X Chen J Liu H. Risk factors predicting posttraumatic hydrocephalus after decompressive craniectomy in traumatic brain injury. World Neurosurg. (2018) 116:e406–13. doi: 10.1016/j.wneu.2018.04.216, 29751189

[54] Romualdo SMF Juratli TA Eyüpoglu I Schackert G Dengl M Prem M . Post-traumatic hydrocephalus after decompressive craniectomy: a multidimensional analysis of clinical, radiological, and surgical risk factors. Neurosurg Rev. (2025) 48:523. doi: 10.1007/s10143-025-03673-0, 40542880 PMC12182525

[55] Kutty RK Sreemathyamma SB Sivanandapanicker J Asher P Prabhakar RB Peethambaran A . The conundrum of ventricular dilatations following decompressive craniectomy: Is ventriculoperitoneal shunt, the only panacea? J Neurosci Rural Pract. (2018) 9:232–9. doi: 10.4103/jnrp.jnrp_395_17, 29725175 PMC5912030

[56] Láng J Ganau M Prisco L Bozsik K Banczerowski P. Syndrome of trephined-underestimated and poorly understood complication after decompressive craniectomy. Ideggyogy Sz. (2016) 69:227–32. doi: 10.18071/isz.69.0227, 29465886

